# Adiponectin Gene Polymorphism (rs17300539) Has No Influence on the Occurrence of Metabolic Syndrome in Women with Polycystic Ovary Syndrome

**DOI:** 10.3390/genes12121902

**Published:** 2021-11-27

**Authors:** Izabela Nowak, Sylwester Ciećwież, Beata Łój, Jacek Brodowski, Agnieszka Brodowska

**Affiliations:** 1 Department of Gynecology, Endocrinology and Gynecological Oncology, Pomeranian Medical University in Szczecin, 71-256 Szczecin, Poland; isabelnowak@hotmail.com (I.N.); agnieszka.brodowska@pum.edu.pl (A.B.); 2 Klinik für Gynäkologie und Geburtshilfe, das Lehrkrankenhaus Sana Hanse Klinikum Wismar, Universität Rostock, 23966 Wismar, Germany; beata.loj@sana.de; 3 Primary Healthcare Center, Pomeranian Medical University, 71-210 Szczecin, Poland; brodoj@pum.edu.pl

**Keywords:** adiponectin, polycystic ovaries, metabolic syndrome, genetic testing, genetic predisposition, screening

## Abstract

Adiponectin (rs17300539) is implicated in the pathogenesis of metabolic syndrome (MS), a common comorbidity of polycystic ovarian syndrome (PCOS). The aim of this study was to analyze the association between adiponectin gene polymorphism and incidence of MS in patients with PCOS. The study included 201 women (age 18 to 35 years), among them 81 patients with PCOS without concomitant MS, 70 subjects with PCOS and concomitant, and 50 regularly menstruating controls. Adiponectin gene polymorphism (11391 G/A, rs17300539) was determined by means of a real-time PCR. The study groups did not differ significantly in terms of their age and frequencies of various genotypes of the adiponectin gene polymorphism. The largest proportion in the whole group was Caucasian women (*n* = 178, 88.56%), who carried the GG genotype of the polymorphism; frequencies of GA and AA genotypes in the whole study group were 10.94% (*n* = 22) and 0.5% (*n* = 1), respectively. The presence of G or A allele of the rs17300539 adiponectin gene polymorphism was not associated with a greater likelihood of PCOS with/without concomitant MS. The hereby presented findings imply that MS is a common comorbidity in women with PCOS. However, the incidence of concomitant MS does not seem to be associated with adiponectin gene polymorphism.

## 1. Introduction

The etiopathogenesis of polycystic ovary syndrome (PCOS) is multifactorial, which is why the syndrome is multifactorial, and its occurrence also depends on ethnic origin and geographical region. PCOS, a common female endocrinopathy, is a current public health challenge and a main cause of anovulatory infertility [1]. Incidence of PCOS varies depending on the used diagnostic criteria, from 6–10% for the National Institutes of Health criteria to 14–17% for the Rotterdam criteria [2]. There is an increased daily production of estrogens and androgens, dependent on LH. It is found in a relationship with the above, also higher, concentrations of testosterone, androstenedione, DHEA, DHEAS, 17-OHP, and estrone in the blood. Therefore, excessive production of lutropin is considered to be the main cause of the disease [3]. Characteristic features of PCOS include oligomenorrhea or amenorrhea, hyperandrogenism, ovarian dysfunction, and ultrasonographic evidence of specific structural abnormalities in the ovaries [4,5]. This division resulted in the fact that in PCOS, we find several phenotypes of women who have similar symptoms, including difficulties in becoming pregnant, but different exposure to diseases [6,7]. Patients with PCOS are also predisposed to metabolic disorders, such as obesity, dyslipidemia, hyperinsulinemia, insulin resistance, diabetes, and metabolic syndrome (MS). Many symptoms are common to both described syndromes (MS and PCOS). Moreover, PCOS is associated with increased risk of endometrial cancer, and probably also breast cancer. Finally, this disease may not infrequently contribute to deterioration of mental health and quality of life.

The excess of white adipose tissue and its accumulation in the visceral compartment are extremely important for the development of the metabolic syndrome. White adipose tissue stores free fatty acids after meals and releases them on an empty stomach. In addition, it secretes substances called adipokines that exhibit paracrine and autocrine effects [8]. As a result of imbalance between esterification and lipolysis of triglycerides, as well as lipogenesis and fatty acid oxidation, adipose tissue dysfunction and obesity develop. Adiponectin improves whole-body energy homeostasis, because it inhibits glucose production in the liver, stimulates fatty acid oxidation in skeletal muscle, and reduces the appetite. When present at an adequate concentration, adiponectin exerts anti-atherosclerotic, antidiabetic, and anti-inflammatory effects, which binds directly to the intracellular regions of AdipoR1 and R2 [9]. The production of endogenous adiponectin is impaired as an effect of obesity and metabolic pathologies. Perhaps a practical therapeutic approach is to use pharmacological or dietary interventions to restore the capacity of adipose tissue in secreting adiponectin [10].

Results of genetic screening imply that metabolic syndrome (MS) is a hereditary condition, inherited in up to 10–30% of cases. This stimulated interest in genetic polymorphisms that may predispose to MS. These inter alia include polymorphisms in leptin, endocannabinoid receptor, peroxisome proliferator-activated receptor γ (PPAR-γ), fat mass and obesity-associated protein (FTO), melanocortin 4 receptor (MC4R), β-3 adrenergic receptor, and the adiponectin gene. Abnormal concentrations of adiponectin may result from polymorphism in its gene. The adiponectin gene (apM1) is located on the long arm of chromosome 3, at 3q27, and its principal regulators are peroxisome proliferators [10]. The 3q27 region, showing a strong link with insulin resistance and type 2 diabetes, has become the subject of many studies in terms of genotypic variability. Mutation in the adiponectin gene may result in a significant decrease in concentration of its target protein, which is associated with increased risk for coronary artery disease, insulin resistance, obesity, type 2 diabetes mellitus, and its complications [11,12,13].

However, the results of published studies analyzing consequences of adiponectin gene polymorphism are inconclusive. Noticeably, most of these studies have been conducted in Asian populations [14,15]. Furthermore, they usually centered around associations between adiponectin gene polymorphism and occurrence of individual components of metabolic syndrome, rather than the risk of MS in general. Additionally, published evidence regarding the genetic link between PCOS and MS is limited. The aim of the study was to determine whether the polymorphism of the adiponectin 11391 G / A gene (rs17300539), which is related to higher body mass index (BMI), an increased risk of insulin resistance, and type 2 diabetes (T2D), influences also to the occurrence of the metabolic syndrome and its components in PCOS patients.

## 2. Materials and Methods

### 2.1. Participants

The study included 201 women in 2012–2016 (age 18 to 35 years) from Western Pomerania province, hospitalized at the Department of Gynecology and Urogynecology, Pomeranian Medical University in Szczecin. The size of the trial for the population of 1,000,000 was calculated by estimating the size of the fraction = 0.5. The confidence level was 95% and the acceptable error margin was 7%, which constituted 196 women. The study subjects were divided into three groups: (1) women with PCOS without concomitant MS (group B1, *n* = 81), (2) women with PCOS and concomitant MS (group B2, *n* = 70), and (3) regularly menstruating women who have been hospitalized due to other reasons (group C, *n* = 50). PCOS was diagnosed on the basis of the Rotterdam criteria from 2003 [16] (https://doi.org/10.1161/CIRCULATIONAHA.109.192644 accessed on 16 September 2021). PCOS was defined, after excluding related disorders, by two of the following three characteristics: (1) oligo- or anovulation, (2) clinical and/or biochemical signs of hyperandrogenism, or (3) polycystic ovaries on an ultrasound image. MS was diagnosed based on the modified criteria of the International Diabetes Federation (IDF) from 2009. Co-occurrence of at least three of the following five deficiencies was required:

(a) Waist circumference >= 80 cm;
(b) Fasting glucose > 100 mg/dL or treatment of type 2 diabetes;
(c) Triglyceride concentration >= 150 mg/dL;
(d) HDL cholesterol < 50 mg/dL;
(e) Elevated blood pressure: systolic >= 130 mmHg and or diastolic >= 85 mmHg or treatment of previously diagnosed arterial hypertension. The study included patients who were not treated pharmacologically due to chronic diseases, following a normal diet. The characteristics of the study group are presented in the table below (Table 1).

### 2.2. Gynecologic Examination and Anthropometry

Each woman was subjected to pelvic exam, ultrasonography of the reproductive organs, and anthropometric measurements (body weight, body height, waist and hip circumferences). Furthermore, blood pressure was measured with the Korotkoff method. Based on their body mass index (BMI) values, the study subjects were classified as presenting with underweight (BMI ≤ 18.49 kg/m^2^), normal weight (BMI 18.5–24.9 kg/m^2^), overweight (BMI 25–29.9 kg/m^2^), grade 1 (BMI 30–34.9 kg/m^2^), grade 2 (35–39.9 kg/m^2^), and grade 3 obesity (BMI ≥ 40 kg/m^2^).

### 2.3. Laboratory Tests

A 5 mL fasting blood sample was collected from each study subject for the purpose of biochemical testing. Fasting venous blood samples were collected between 7:00 AM and 9:00 AM after 12 h overnight fast. All tests were conducted immediately after collection of the blood. Serum concentrations of total cholesterol, triglycerides, LDL and HDL cholesterol were determined with an enzymatic-colorimetric method using the Cobas^®^ 4000 analyzer series (Roche Diagnostics, Basel, Switzerland, 2008). Furthermore, the oral glucose tolerance test was performed on each study subject with the determination of baseline fasting plasma concentrations of glucose and insulin (Ins0′), followed by measurement of these parameters 120 min after drinking 75.0 g anhydrous glucose dissolved in 250–300 mL of water. Plasma concentration of glucose was determined with an enzymatic method with hexokinase, and plasma level of insulin by means of electrochemiluminescence.

### 2.4. Genetic Testing

**Figure 1:**
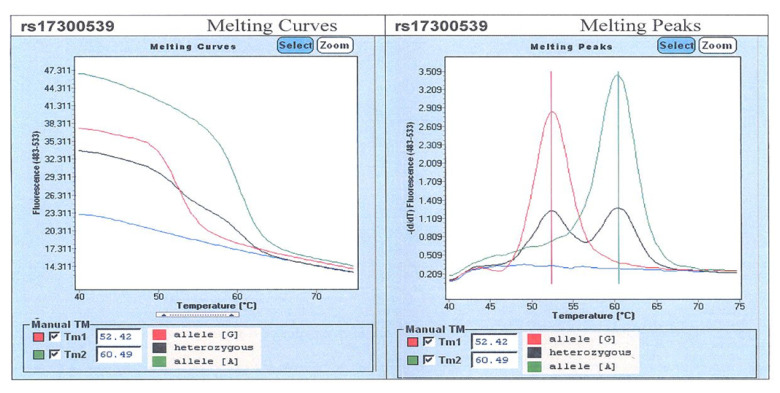
Analysis of the melting curves for individual alleles of ADIPOQ gene polymorphism (rs17300539).

Additionally, 5 mL whole blood sample was obtained from each study subject to determine 11391 G/A (rs17300539) adiponectin gene polymorphism. Upon isolation of DNA with the aid of Invisorb Spin Blood Midi Kits, genetic polymorphisms were determined by means of a real-time PCR on LightCycler II device (Roche Diagnostics, Basel, Switzerland, 2008), on the basis of melting curves for individual alleles (Figure 1). The melting curves for the G allele were Tm = 52.42 °C, and for A allele, Tm = 60.49 °C. Real-time PCR comprised the following stages: initial denaturation, 3-stage PCR (denaturation, primer binding, DNA replication), melting and cooling.

### 2.5. Statistical Analysis

Normal distribution of continuous variables was verified with the Kolmogorov–Smirnov test. Statistical characteristics of continuous variables are presented as arithmetic means and standard deviations. The statistical significance of differences between two groups was verified with the Mann–Whitney U test, and the significance of differences between multiple groups was verified with analysis of variance (ANOVA) or the Kruskal–Wallis test. The likelihood of PCOS and MS in carriers of various genotypes of adiponectin gene polymorphism was estimated with a logistic regression model, on the basis of odds ratios (ORs) and their 95% confidence intervals (95%CIs). Statistical significance of relationships within the model was verified with Pearson’s chi-squared test or a two-tailed Fisher’s exact test. The results of all tests were considered significant for *p*-values < 0.05, and at a threshold of statistical significance for *p*-values between 0.051 and 0.099. All statistical calculations were carried out with STATA 11 package (license No. 30110532736).

## 3. Results

Mean age of the study subjects was 27.92 years (range 18–35 years). The study groups did not differ significantly in terms of their age (Table 1).

The highest BMI value was found in the B2 group, as was expected. The average value of body mass index in that group was 31.5, which means the 1st class of obesity. Waist circumference was the largest also in group B2. The BMI values in the other group were similar, as was the fasting blood glucose value. Worth noting is the value of triglycerides, which turned out to be the lowest in the B1 group.

The largest proportion of the study subjects (*n* = 178, 88.56%) carried the GG genotype of rs17300539 adiponectin gene polymorphism; frequencies of GA and AA genotypes in the whole study group were 10.94% (*n* = 22) and 0.5% (*n* = 1), respectively. Irrespective of the study group, the distributions of various genotypes were consistent with the Hardy–Weinberg equilibrium. The study groups did not differ significantly in the frequencies of various genotypes of rs17300539 adiponectin gene polymorphism (Table 2).

Presence of G or A allele of the rs17300539 adiponectin gene polymorphism was not associated with a greater likelihood of PCOS with/without concomitant MS (Table 3).

## 4. Discussion

In this study, 46.35% of patients with PCOS were diagnosed with concomitant MS in line with the IDF diagnostic criteria from 2009. Published data on the incidence of MS in women with PCOS are inconclusive. These discrepancies may reflect confounding effects of other factors, such as BMI, age, diet, and/or criteria used to diagnose PCOS and MS [17]. Incidence of MS may vary depending on the cut-off values used to diagnose individual components of the syndrome. In turn, an important determinant of PCOS incidence is its phenotype according to the Rotterdam criteria [18,19]. According to the literature, the risk of concomitant metabolic disorders is particularly high in patients with the A phenotype of PCOS, i.e., with oligo ovulation, hyperandrogenism, and polycystic ovaries [18].

Published evidence suggests that the incidence of MS in patients with polycystic ovaries is 2- to 4-fold higher than in the general population of healthy reproductive-age women [20,21]. However, available data on the incidence of MS vary greatly depending on subjects’ ethnicity and country of residence, from 8.2% in southern Italy [22] to 14.5% in Korea [23], 28.4% in Brazil [24], 37.9% in India [25], and up to 43–46% in the United States [26]. Moreover, the degree of gene expression largely depends on environmental factors and diet [27,28,29].

Our study groups were relatively homogeneous in terms of age, with mean age ranging from 27.17 years for patients with PCOS to 28.39 years for subjects with PCOS and MS, and to 28.48 years for healthy controls. This observation is consistent with the evidence from some clinical studies in which the risk of metabolic disorders in women with PCOS was shown to be independent of age [22,30].

The genetic background of MS has been within the scope of researchers’ interest for years. First-degree relatives of women with PCOS, including their brothers and fathers, were shown to be at increased risk of MS [31]. Furthermore, Trottier et al. [32] demonstrated that daughters and sisters of patients with PCOS are predisposed to the development of insulin resistance at a very young age. This observation is consistent with the results published previously by Sir-Petermann et al. [33], according to whom female first-degree relatives of women with PCOS are prone to metabolic disorders well before puberty. MS was postulated to be associated with polymorphism in some genes, among them adiponectin gene polymorphism. Sun X and others confirmed the association of rs17300539 polymorphism in the adiponectin gene and PCOS in the Chinese population. According to the research results they even suggest that the above-mentioned polymorphism could be a risk marker of PCOS. In particular, the rs17300539 AA genotype seems to have significant influence on predisposition to PCOS occurrence [34]. However, although this polymorphism and its consequences were a subject of many previous studies, we still lack conclusive evidence in this matter.

The vast majority of subjects participating in our study carried the GG genotype for the 11391 G>A adiponectin gene polymorphism. This genotype was found in 90% of healthy controls, in 87.65% of patients with PCOS, and in 88.57% of subjects in whom PCOS coexisted with MS. In turn, the GA genotype of the analyzed polymorphism was found in 10% of women from the control group, as well as in 10% and 12.35% of patients with PCOS with and without concomitant MS, respectively. Only one woman, a patient with PCOS and concomitant MS, carried the AA genotype for adiponectin gene polymorphism. We did not find a statistically significant association between the presence of G or A alleles of adiponectin gene polymorphism and the likelihood of concomitant MS [35].

To the best of our knowledge, none of the previous studies analyzed adiponectin gene polymorphism is such selected groups of patients. According to the literature, the penetrance of specific genotypes of this polymorphism is country- and ethnicity-specific. In a French population of 4500 randomly selected female and male Caucasians, the distribution of genotypes was similar to that observed in our series: 82% of the probands carried the GG genotype, whereas GA and AA genotypes were found in 16.9% and 0.9% of the study subjects, respectively [35]. In an Iranian study [36], including 238 patients with type 2 diabetes mellitus and 159 healthy subjects, the GG genotype was found in 67.6% of the diabetics and in 25.4% of the controls, and the GA + AA genotype was found in 4.9% and 2.1%, respectively. A Croatian study [37] of 149 young unrelated subjects with MS and obesity identified 59.7% of GG genotype carriers, along with 38.3% and 2% of subjects with GA and AA genotypes, respectively. Finally, GG was the only genotype of the adiponectin gene polymorphism found in 867 Korean women between 20 and 69 years of age [38].

One potential limitation of this study is the relatively small sample size enforced by the high cost of genetic tests. Consequently, our hereby presented findings should be considered as preliminary results and a starting point for future research on the genetic background of MS observed during the course of PCOS.

## 5. Conclusions

The hereby presented findings imply that MS is a common comorbidity in women with PCOS. However, the incidence of concomitant MS does not seem to be associated with rs17300539 adiponectin gene polymorphism. Our study was limited by a small sample. More research is needed to investigate the association between other SNPs of the adiponectin gene, MS, and PCOS.

## Figures and Tables

**Table 1:** The characteristics of the study group.

	C (*n* = 50) Mean ± SD	B1 (*n* = 81) Mean ± SD	B2 (*n* = 70) Mean ± SD	P C vs. B1	P C vs. B2	P B1 vs. B2
Age (year)	28.48 ± 3.88	27.17 ± 4.14	28.39 ± 4.91	0.655	0.991	0.125
BMI (kg/m^2^)	21.81 ± 1.63	22.52 ± 4.08	31.52 ± 6.46	0.068	0.000	0.000
WC (cm)	73.26 ± 5.33	76.10 ± 9.98	94.64 ± 12.71	0.079	0.000	0.000
GLU (mg/dL)	88.89 ± 8.26	89.74 ± 8.55	94.34 ± 10.43	0.151	0.000	0.000
TGL (mg/dL)	112.89 ± 20.81	75.26 ± 29.27	145.61 ± 63.21	0.000	0.000	0.000
HDL (mg/dL)	58.51 ± 8.94	66.67 ± 22.84	47.82 ± 12.67	0.000	0.000	0.000
SBP (mmHg)	115.60 ± 8.60	112.85 ± 11.42	136.00 ± 10.70	0.980	0.000	0.000
DBP (mmHg)	72.22 ± 7.31	70.60 ± 9.66	85.54 ± 8.80	0.315	0.000	0.000

SD—standard deviation, C—normally menstruating controls, B1—women with PCOS without MS, B2—women with PCOS and MS, *n*—number, P—*p*—value, WC—waist circumference, GLU—glucose, TGL—triglycerides, HDL—high density lipoprotein, SBP—systolic blood pressure, DBP—diastolic blood pressure.

**Table 2:** Frequencies of various genotypes of the rs17300539 adiponectin gene polymorphism in the study groups.

Genotype	K (*n* = 50)	B1 (*n* = 81)	B2 (*n* = 70)	Overall
GG	*n* = 45 (90.00%)	*n* = 71 (87.65%)	*n* = 62 (88.57%)	*n* = 178
GA	*n* = 5 (10.00%)	*n* = 10 (12.35%)	*n* = 7 (10.00%)	*n* = 22
AA	*n* = 0 (0.00%)	*n* = 0 (0.00%)	*n* = 1 (1.43%)	*n* = 1
Chi-square	2.13	df = 4	*p* = 0.71118	

K—normally menstruating controls, B1—women with PCOS without MS, B2—women with PCOS and MS, df—degrees of freedom.

**Table 3:** Associations between presence of G or A allele of rs17300539 adiponectin gene polymorphism and the likelihood of PCOS with/without concomitant MS; results of logistic regression analysis.

Groups	Genotype	OR	95%CI	*p*
B1 vs. K	GG	0.79	0.25–2.46	0.683
B1 vs. K	GA	1.27	0.41–3.95	0.683
B2 vs. K	GG	0.86	0.26–2.81	0.804
B2 vs. K	GA	1.00	0.30–3.35	1.000
B2 vs. B1	GG	1.09	0.41–2.94	0.862
B2 vs. B1	GA	0.79	0.28–2.20	0.650

OR—odds ratio, 95% CI—95% confidence interval, K—normally menstruating controls, B1—women with PCOS without MS, B2—women with PCOS and MS.
